# The Novel Integration of a Learning Community Model In Older Adult Workforce Development Research

**DOI:** 10.1093/geroni/igaf122.2503

**Published:** 2025-12-31

**Authors:** Jennifer Crittenden, Mary Lou Ciolfi, Lenard Kaye, Rachel Coleman, Richa Arya, Catherine Taylor

**Affiliations:** University of Maine Center on Aging, Orono, Maine, United States; UMaine Center on Aging, Orono, Maine, United States; University of Maine, orono, Maine, United States; University of Maine Center on Aging, Orono, Maine, United States; University of Maine, Bangor, Maine, United States; University of Maine, Bangor, Maine, United States

## Abstract

Learning communities foster sharing of knowledge and skills in educational and workplace settings but are infrequently used in research. An AmeriCorps Seniors-funded research project at the University of Maine Center on Aging focused on older adult workforce development programs (WDPs) established a learning community (LC) to identify lessons learned and provide peer support and education across seven WDPs. Early findings shed light on the opportunities and challenges of implementing an LC model within a research initiative. Located across five U.S. states, LC members meet monthly via Zoom for discussions, small group activities, and guest speakers. Integration of the LC in the research process has yielded substantial insight into the challenges facing older adult WDPs. Challenges noted by participants include: 1) building relationships and trust between members, primarily attributed to the online modality making it difficult to form meaningful connections; 2) organizational stressors (e.g., high turnover of WDP staff, lack of time for meaningful participation); and 3) concern about program performance monitoring which may hamper candid communication. LC model adaptation and creativity have been key for overcoming these challenges. Incorporating short interactive activities at the start of meetings and regular program updates by participants with structured time for sharing and mutual assistance has facilitated relationship building, as has the presence of LC staff “champions”. Learning communities can provide opportunities for growth and development for program participants and valuable insight for researchers. Responsive and adaptive strategies can increase engagement and effectiveness when using an LC in research contexts using online modalities.

